# Comparison of the Correlation Between Cerebral [^18^F]FDG Metabolism as Assessed by Two Asymmetry Indices and Clinical Neurological Score in Patients with Ischemic Cerebrovascular Disease

**DOI:** 10.1007/s11307-025-02002-7

**Published:** 2025-04-15

**Authors:** Yuxin Liang, Bixiao Cui, Linlin Ye, Bin Yang, Yi Shan, Hongwei Yang, Lei Ma, Miao Zhang, Jie Lu

**Affiliations:** 1 Department of Radiology and Nuclear Medicine, Xuanwu Hospital Capital Medical University, Changchun Steel 45 #, Beijing, 100053 Xicheng District China; 2 Key Laboratory of Magnetic Resonance Imaging and Brain Informatics, Changchun Steel 45 #, Beijing, 100053 Xicheng District China; 3 Department of Rehabilitation, Xuanwu Hospital, Capital Medical University, Beijing, 100053 China; 4 Department of Neurosurgery, Xuanwu Hospital Capital Medical University, Beijing, 100053 China

**Keywords:** Ischemic cerebrovascular disease, Asymmetry index, Positron emission tomography, Brain glucose

## Abstract

**Purpose:**

Two types of asymmetry index (AI) have been utilized in evaluating cerebral function in ischemic cerebrovascular disease, however, few data exist on the differences between these AI measures. This study aimed to compare the two AIs in assessing PET cerebral metabolism and their correlation with clinical scales, to explore their potential value and applications in clinical settings.

**Procedures:**

Seventy patients diagnosed with subacute and chronic ischemic stroke were retrospectively analyzed. All patients underwent 2-deoxy- 2-[^18^F]fluoro-D-glucose ([^18^F]FDG) PET/MR scans and were assessed using the National Institutes of Health Stroke Scale (NIHSS) and the Modified Rankin Scale (mRS). Nineteen patients underwent a repeat [^18^F]FDG PET/MR scan one year later. Two voxel-wise AI methods, designated as AI_1_ and AI_2_, were calculated based on standardized uptake value ratio (SUVR). The hypometabolism on affected side assessed by different AI methods were compared. The correlations between the hypometabolism and the clinical scores were analyzed.

**Results:**

The volume and percentage of decreased [^18^F]FDG metabolism assessed by AI_2_ was larger than that obtained from AI_1_ (all *p* < 0.0001). The correlation coefficients between the clinical scores and the decreased metabolism in temporal and parietal lobes assessed by AI_1_ method were all higher than those from AI_2_. In addition, the improved follow-up patients showed more pronounced metabolic improvement as assessed by AI_1_.

**Conclusions:**

The assessment of cerebral [^18^F]FDG metabolism in patients with unilateral internal carotid/middle cerebral artery steno-occlusion to reflect clinical neurological function using the AI_1_ method demonstrated superior performance in comparison to the AI_2_ method.

**Supplementary Information:**

The online version contains supplementary material available at 10.1007/s11307-025-02002-7.

## Introduction

Ischemic cerebrovascular disease (ICVD) is predominantly responsible for the majority of strokes, characterized by progressive steno-occlusive changes at the terminal portion of the internal carotid artery (ICA) or middle cerebral artery (MCA) [1]. As one of the leading causes of death and disability worldwide, ICVD has posed a huge burden in public health issue of growing importance [2]. The risk of stroke recurrence among these patients remains high [3], which has prompted us to contemplate effective long-term monitoring to control the risk of reinfarction in patients with cerebral infarction.

Brain is an organ with high metabolic activity that primarily utilizes glucose as its source of energy. The 2-deoxy- 2-[^18^F]fluoro-D-glucose ([^18^F]FDG) serves as a good marker revealing the in distribution of glucose uptake by cells [4, 5]. The hybrid [^18^F]FDG PET/MR allows for simultaneously acquire precise structural data with excellent tissue contrast and metabolic information [6]. Studies related to cerebral metabolism suggest that the increased metabolism observed in the peri-infarct region of acute stroke patients may suggest the presence of surviving tissue that can be salvaged by reperfusion, whereas significant metabolic reductions have been found in the peri-infarct region of patients with chronic ischemic cerebrovascular lesions [7, 8]. From a pathophysiological point of view, the cerebral hypometabolism in of ICVD patients reflects reduced brain cell activity and further reflects changes in brain function [9]. Evaluating the cerebral hypometabolism in patients with unilateral subacute and chronic ICVD of the affected side brain tissue relative to the normal side brain tissue helps to reflect the objective situation of disease. Quantifying hypometabolism by evaluating the asymmetrical change of brain tissue metabolism potential value for monitoring their clinical outcomes and recovery effects [10, 11]. By capturing the relative metabolic deficit, the asymmetry index (AI) is especially suited to quantify the feature of imbalance.

AI is commonly employed for assessing the distribution asymmetry of cerebral metabolism or cerebral blood flow [12]. Previous studies on functional brain changes in ICVD patients have shown that the AI values for glucose metabolism were significantly lower after effective treatment [13]. Van Niftrik CHB et al. [14] using an AI method demonstrated a robust association between the degeneration after stroke and the ipsilateral thalamic diaschisis, while Zhu Y et al. [15] found the metabolic assessment of another AI method was correlated with the predisposing factors in ICVD. However, the results of different AI methods may yield disparate insights into the clinical neurologic status of a given subject. It remains a question worthy of further exploration as to which AI computational approach is better suited to assessing decreased [^18^F]FDG metabolism and facilitating comprehension of alterations in disease state among ICVD patients.

This study incorporated both methods reflecting the relative differences between the two sides of brain metabolism, AI_1_ and AI_2_, and systematically compared their clinical applicability in subacute or chronic unilateral ICVD. The aim of this study was to provide a framework for methodological selection of AI-based brain metabolism assessment and to provide a reference basis for the prediction of patients'long-term prognosis, thus supporting clinical stratified management and individualized therapeutic decision-making.

## Materials and Methods

### Subjects

**Fig. 1:**
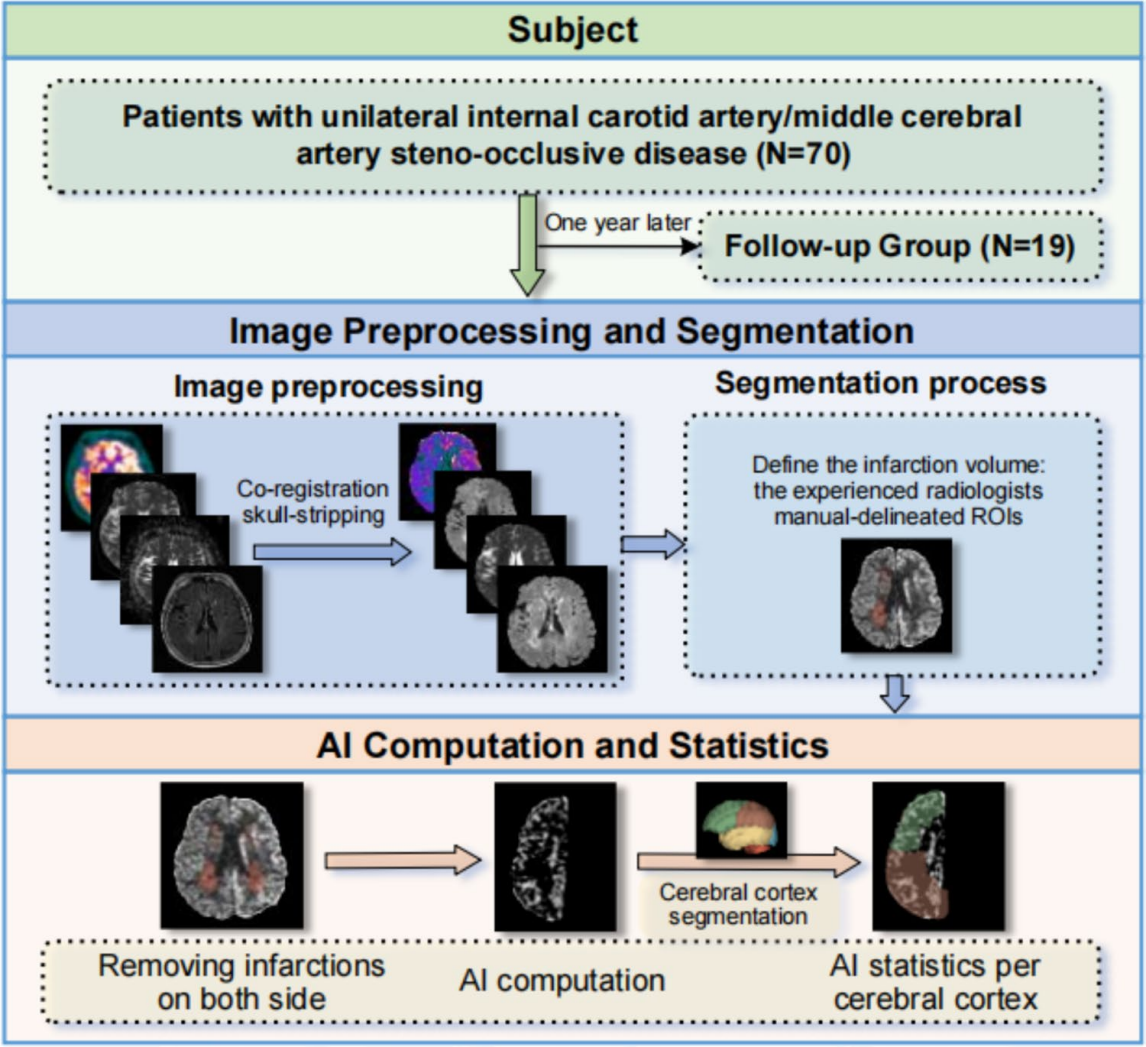
Flowchart of the study design. AI, asymmetry index. ROIs, regions of interest

Seventy patients with subacute and chronic ischemic stroke in Xuanwu Hospital, Capital Medical University from March 2018 to August 2023 were retrospectively screened and included according to the following criteria: (1) a confirmed diagnosis of ICVD due to ICA or MCA steno-occlusive; (2) a history of a clinically confirmed stroke of the relevant ICA or MCA territory; and (3) consecutive PET/MR, DWI, computed tomography angiography and magnetic resonance angiography scans. The exclusion criteria were: (1) presence of multiple infarcts on both of the cerebral hemisphere; (2) other neurological disorders that can cause abnormal brain metabolism; and (3) the contraindication for MRI or artefacts on MRI. Following a year of rehabilitation training (non-operative treatment), nineteen patients underwent a repeat follow-up [^18^F]FDG PET/MR scan. The infarcts size and the stroke severities were not used as the criteria for screening patients in this study. The neurological function was assessed using the National Institutes of Health Stroke Scale (NIHSS) and the Modified Rankin Scale (mRS) scores on admission to hospital. This study was approved by the ethical approval institutional review board of Xuanwu Hospital, Capital Medical University and conducted in accordance with the Declaration of Helsinki. The written informed consents were obtained from all participating patients. Figure 1 shown the flowchart of the study design.

### PET/MR Image Acquisition

All scans were collected on a hybrid time of flight (TOF) PET/MR system (Signa, GE Healthcare). Before examination, all subjects fasted for minimum of 6 h and blood glucose levels were checked to ensure a glycemic level below 8 mmol/L. [^18^F]FDG (3.7 MBq/kg) were injected manually in the median cubital vein, the PET/MR scan started at 50 min post-injection. A 19-channel head and neck union coil was used to achieve a high signal-to-noise ratio for PET/MR imaging. Subjects were placed in a supine position to ensure being in the center of the field in view and were instructed to remain calm with their eyes closed.

The [^18^F]FDG PET images were obtained over a period of 10 min. The PET data were corrected for attenuation, scatter, random, decay, and dead time. The default attenuation correction sequences (Dixon MR sequences) and MR scans were simultaneously obtained. The Dixon MR sequences was automatically prescribed and acquired as follows: LAVA-Flex (GE Healthcare) axial acquisition, repetition time (TR) = 4 ms, echo time (TE) = 1.7 ms, slice thickness = 5.2 mm, 120 slices, pixel size = 1.95 × 2.93 mm^2^, and acquisition time = 18 s. The corrected PET data were reconstructed using a time-of-flight, point spread function, ordered subset expectation maximization (time of flight—point spread function—office of systems engineering and management, TOF-PSF-OSEM) algorithm with 8 iterations and 32 subsets, and a 3 mm cut-off filter. The resulting pixel size was 1.82 × 1.82 × 2.78 mm^3^.

PET and MR imaging data were simultaneously acquired. The main MRI sequences included the T2 fluid-attenuated inversion recovery (T2-FLAIR) sequence (voxel size = 0.94 × 0.94 × 4.00 mm^3^, TR = 11,000 ms, TE = 141 ms, and slices = 32), the diffusion-weighted image (DWI) (b = 0/1000) sequence (voxel size = 1.88 × 1.88 × 4.00 mm^3^, TR = 6189 ms, TE = 74.7 ms, and slices = 32).

### PET/MR Image Preprocessing

The PET/MR image preprocessing using 3D Slicer Tool (version 5.5.0, https://www.slicer.org). Firstly, the skull-stripping was performed on all MRI sequences (T2-FLAIR, DWI, ADC) as well as [^18^F]FDG PET by using HD-BET brain extraction toolkit algorithm [16]. Subsequently, each subject’s [^18^F]FDG PET image was performed partial volume correction (PVC) by using the Van-Cittert algorithm, and the standardized uptake value ratio (SUVR) was calculated using the pons as the reference region [17, 18]. Then, the DWI (b = 1000), ADC, and PET images were all co-registered to the individual T2-FLAIR image using the General Registration BRAINS algorithm.

### Segmentation Process

Two experienced neuroradiologists independently and manually delineated the cerebral infarction regions based on the T2-FLAIR sequence. For cases of disagreement, a consensus was reached in a separate session. The individual brain PET image was subdivided into 83 regions by using an automated labeling system, neuroparc (https://github.com/neurodata/neuroparc) [19]. And the bilateral frontal, temporal, parietal, and occipital regions were left behind in preparation for the next step of analysis.

### Asymmetry Index (AI) Measurements

AI is used to measure the asymmetry of a distribution. [^18^F]FDG PET images were converted into maps that represented the standardized uptake value ratio (SUVR) for each voxel. SUVR was calculated by dividing the tissue concentration of radioactivity (kBq/mL) in the region of interest by the mean activity concentration in a reference region. AI was calculated in the individual brain map after removing all cerebral infarctions on both ipsilateral and contralateral sides to assess the left–right asymmetry on SUVR value. Two formulas for evaluating asymmetry, named AI_1_ and AI_2_, were computed based on the following two Eqs. (1) (2) respectively [20]:

1 $$A{I}_{1}=\frac{SUV{R}_{contralateral}-SUV{R}_{ipsilateral}}{SUV{R}_{contralateral}}\times 100\%$$

2 $$A{I}_{2}=\frac{2\times \left(SUV{R}_{contralateral}-SUV{R}_{ipsilateral}\right)}{SUV{R}_{contralateral}+SUV{R}_{ipsilateral}}\times 100\%$$

where contralateral (ipsilateral) represents the affected side (unaffected side). Subsequently, the volume of decreased metabolism on affected side was defined as those voxel-wise AI value higher than 10% [21]. The percentage change in frontal, temporal, parietal, occipital regions and cerebral hemisphere on affected side compared to the unaffected side was calculated respectively.

### Statistical Analysis

All statistical analyses were conducted using IBM SPSS Statistics for Windows, version 27.0 (IBM). Categorical variables were expressed as percentages. Normally distributed metric variables were expressed as mean ± standard deviation (SD). Non-normally distributed variables were expressed as median (range). Bland–Altman analysis was used for analyzing the quantitative agreement between AI_1_ and AI_2_ methods. Metabolic differences across the AI calculation methods were assessed using Paired T-tests. The statistical significance was determined at *p* value < 0.05. The correlation between the AI assessments and NIHSS/mRS score were analyzed using Spearman’s rank correlation. The data before and after the follow-up were all analyzed.

## Results

### Patient Characteristic

**Fig. 2:**
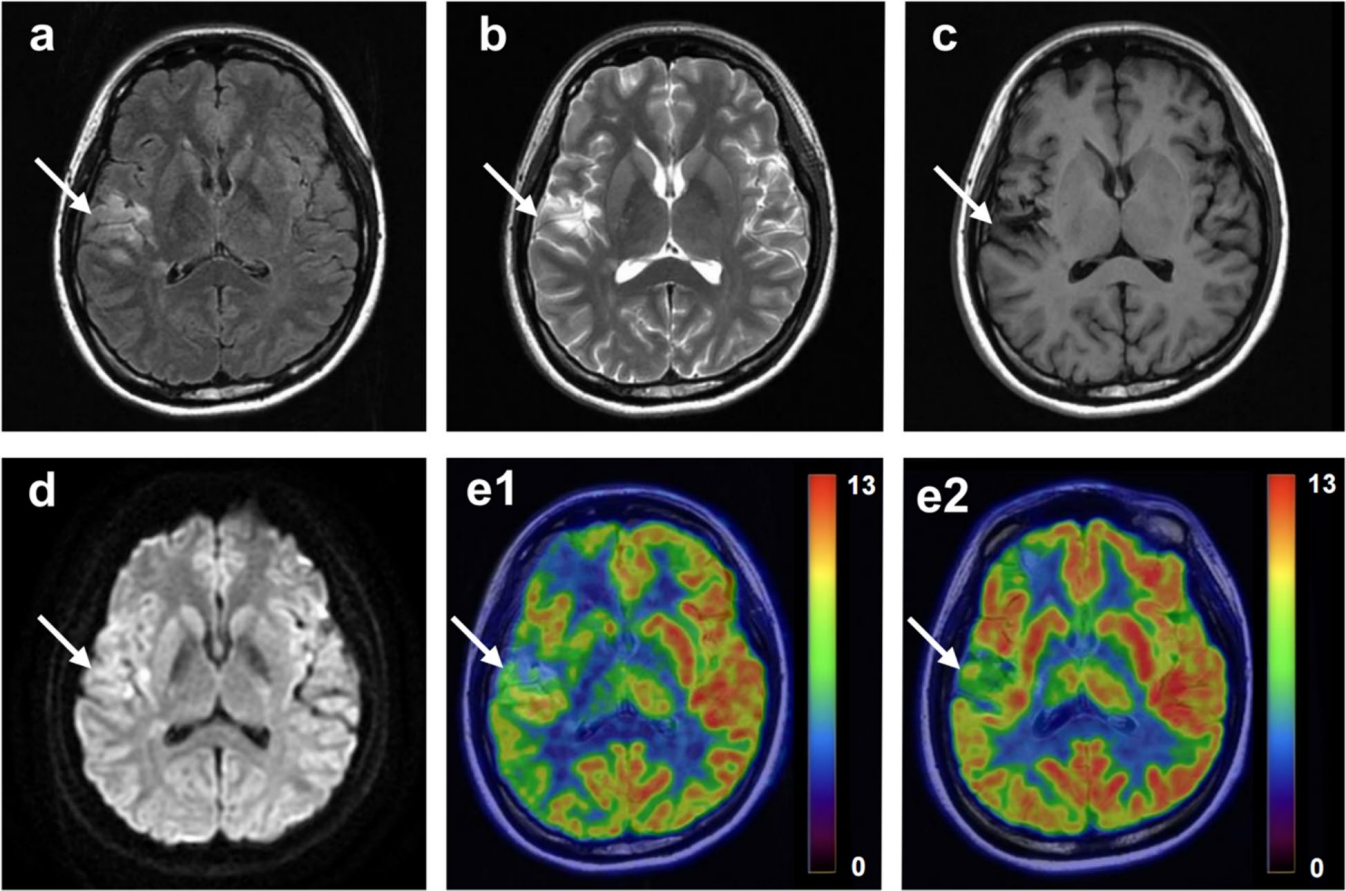
Example of a participate with ischemic cerebrovascular disease. A 24-year-old woman with ischemia in the right hemisphere. The NIHSS and mRS scores improved from 5 and 4 before follow-up to 1 and 2, respectively. On the T1 **(a)**, T2 **(b)**, T2-FLAIR **(c)**, and DWI **(d)**, the infarction can be seen in the right hemisphere. On the [^18^F]FDG PET-MR in the pre-follow-up period **(e1)**, the metabolic impairment can be seen in the peri-infarct area. On the [^18^F]FDG PET-MR in the post-follow-up period **(e2)**, the hypometabolic state of the infarct surrounding areas in the right cerebral hemisphere exhibited an improvement from the previous state

A total of 70 patients, comprising 51 males (72.86%) with a mean age of 52 ± 11 years, presenting with unilateral internal carotid artery and middle cerebral artery steno-occlusive disease, underwent a [^18^F]FDG PET/MR scan. The NIHSS and mRS scores were recorded for each patient. Additionally, nineteen of these patients (including 10 males (52.63%) with a mean age of 51 ± 14 years at pre-follow-up and 53 ± 13 years at post-follow-up) underwent a repeated follow-up [^18^F]FDG PET/MR scan and were reassessed using the NIHSS and mRS after a year of rehabilitation training. Figure 2 illustrates the exemplary imaging of one participant both before and after the follow-up period. Table 1 showed the detailed demographic characteristics.

**Table 1:** Demographic characteristics of participants

	Unilateral internal carotid artery/middle cerebral artery steno-occlusive disease patients			
	All (N = 70)	Pre-follow-up (N = 19)	Post-follow-up (N = 19)	*p* value
Gender (M/F)	51/19	10/9		
Age (years)	52.36 ± 10.57	51.32 ± 13.64	52.95 ± 13.43	0.2783
NIHSS	3 (0, 14)	5 (1, 10)	2 (0, 9)	0.0047
mRS	2 (0, 5)	4 (1, 4)	2 (0, 4)	< 0.0001
Blood glucose (mmol/L)	5.92 ± 1.00	5.85 ± 1.16	5.92 ± 1.24	0.1163
Injection (MBq)	306.18 ± 52.16	309.25 ± 61.04	294.12 ± 50.47	0.1056

Gender was expressed as percentage. Age, blood glucose, and injection were expressed as mean ± standard deviation. The NIHSS and mRS scores were expressed as median (range). The p value was shown for the statistically significant difference between pre-follow-up and post-follow-up. NIHSS, National Institutes of Health Stroke Scale; mRS, Modified Rankin Scale

### The Hypometabolism on Affected Side as Assessed by AI_1_ and AI_2_

We found the hypometabolic volume and percentage in individual lobes and entire hemisphere on affected side (without infarction area) obtained from AI_2_ method were all greater than that of AI_1_ (all *p* < 0.0001) (shown in Table 2). Bland–Altman analysis showed a close degree of agreement between measurements of hypometabolism between AI_1_ and AI_2_ methods (Suppl. Figure 1).

**Table 2:** The hypometabolic volume and percentage in participants (N = 70)

	Volume (ml)				Percentage (%)		
Region	AI_1_	AI_2_	*p* value		AI_1_	AI_2_	*p* value
FL	50.50 ± 12.45	51.01 ± 12.46	< 0.0001		45.25 ± 10.72	45.71 ± 10.69	< 0.0001
TL	41.31 ± 10.98	41.84 ± 11.02	< 0.0001		42.36 ± 11.13	42.90 ± 11.11	< 0.0001
PL	64.43 ± 15.69	65.14 ± 15.74	< 0.0001		44.25 ± 9.29	44.74 ± 9.26	< 0.0001
OL	20.43 ± 7.77	20.69 ± 7.86	< 0.0001		40.22 ± 9.65	40.73 ± 9.66	< 0.0001
Affected hemisphere	176.66 ± 36.09	178.68 ± 36.11	< 0.0001		43.52 ± 8.72	44.02 ± 8.69	< 0.0001

Mean ± standard deviation was shown for the regional hypometabolic volume and percentage evaluated by AI (AI_1_, AI_2_). The p value was shown for the statistically significant difference between AI_1_ and AI_2_. FL, Frontal lobe; TL, Temporal lobe; PL, Parietal lobe; OL, Occipital lobe

### Correlation Between the Hypometabolic Volume as Evaluated by AI (AI_1_, AI_2_) and the NIHSS/mRS Score

**Fig. 3:**
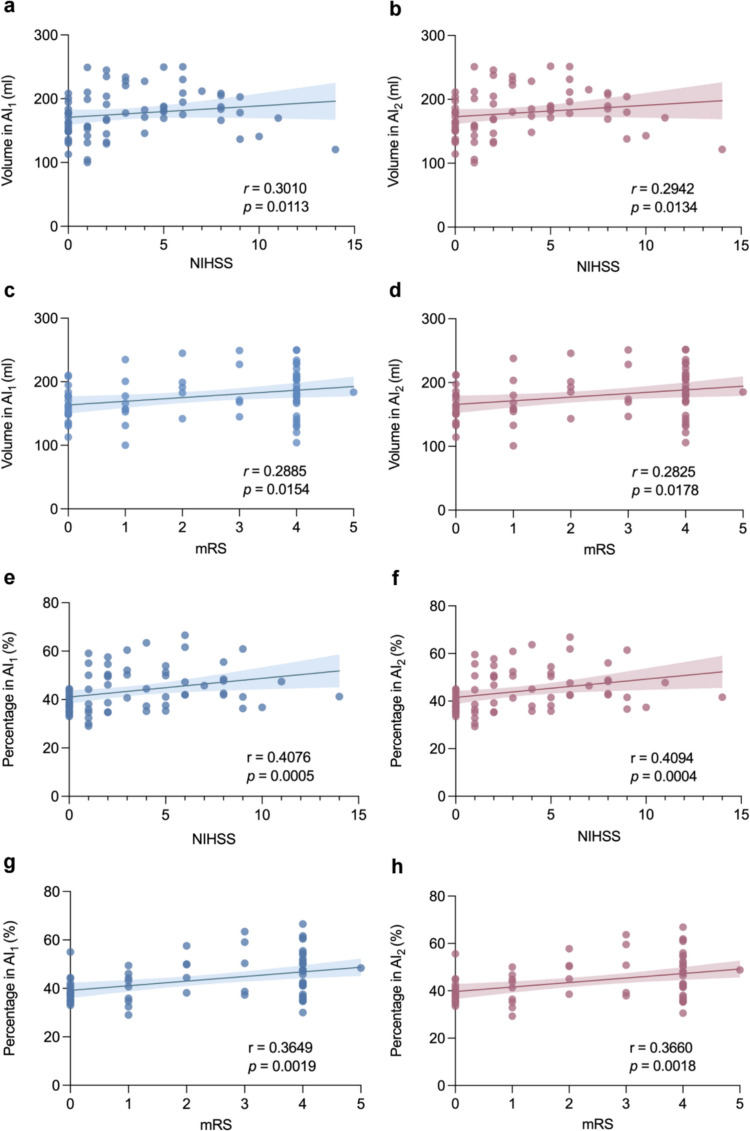
The correlation between the hypometabolic volume and percentage in AI_1_ and AI_2_ with the clinical scores(NIHSS/mRS) (N = 70). Percentage was presented as the ratio of the hypometabolic volume in affected hemisphere. NIHSS, National Institutes of Health Stroke Scale. mRS, Modified Rankin Scale. **(a-b)** Correlation between the volume and NIHSS score. **(c-d)** Correlation between the volume and mRS score. **(e–f)** Correlation between the percentage and NIHSS score. **(g-h)** Correlation between the percentage and mRS score

A significant correlation between the hypometabolic volume (without infarction area) and the NIHSS score can be observed while the correlation coefficients obtained from the AI_1_ method were all higher than those from the AI_2_ (Fig. 3 and Suppl. Figure 2). The correlation coefficients in temporal lobe obtained by the AI_1_ and AI_2_ methods were 0.3403 (*p* = 0.0039) and 0.3393 (*p* = 0.0041) (Suppl. Figure 2a-b) while in parietal lobe were 0.3076 (*p* = 0.0096) and 0.3052 (*p* = 0.0102) (Suppl. Figure 2e-f), respectively. And the correlation coefficients in whole affected hemisphere obtained by the AI_1_ and AI_2_ methods were 0.3010 (*p* = 0.0113) and 0.2942 (*p* = 0.0134) (Fig. 3a-b), respectively.

Similar results demonstrated that the hypometabolic volume (without infarction area) obtained by the AI_1_ exhibited a stronger correlation with mRS than that obtained from AI_2_ (Fig. 3 and Suppl. Figure 2). The correlation coefficients in temporal lobe obtained by the AI_1_ and AI_2_ methods were 0.2751 (*p* = 0.0212) and 0.2745 (*p* = 0.0215) (Suppl. Figure 2c-d) while in parietal lobe were 0.2885 (*p* = 0.0154) and 0.2825 (*p* = 0.0178) (Suppl. Figure 2 g-h), respectively. And the correlation coefficients in whole affected hemisphere obtained by the AI_1_ and AI_2_ methods were 0.3010 (*p* = 0.0113) and 0.2942 (*p* = 0.0134) (Fig. 3c-d), respectively.

### Correlation Between the Hypometabolic Percentage as Evaluated by AI (AI_1_, AI_2_) and the NIHSS/mRS Score

The significant correlation between the hypometabolic percentage (without infarction area) and the NIHSS score can be observed while the correlation coefficients obtained from the AI_1_ method were all higher than those from the AI_2_ (Suppl. Figure 3 and Suppl. Figure 4). The correlation coefficients in temporal lobe were 0.3550 (*p* = 0.0026) and 0.3516 (*p* = 0.0028) (Suppl. Figure 3e-f) while in parietal lobe were 0.4887 (*p* < 0.0001) and 0.4866 (*p* < 0.0001) (Suppl. Figure 4a-b), respectively.

The higher correlation in AI_1_ could also be found between the hypometabolic percentage and the mRS score (Suppl. Figure 3 and Suppl. Figure 4). The correlation coefficients in temporal lobe obtained by the AI_1_ and AI_2_ methods were 0.2917 (*p* = 0.0143) and 0.2874 (*p* = 0.0159) (Suppl. Figure 3 g-h) while in parietal lobe were 0.4504 (*p* < 0.0001) and 0.4485 (*p* < 0.0001) (Suppl. Figure 4c-d), respectively.

The hypometabolic percentage in whole affected hemisphere as assessed by AI_2_ method demonstrated a slightly stronger correlation with NIHSS/mRS scores than that obtained from AI_1_ (Fig. 3). Specifically, the correlation coefficients with NIHSS score obtained by the AI_1_ and AI_2_ methods were 0.4076 (p = 0.0005) and 0.4094 (p = 0.0004) (Fig. 3e-f) while with mRS score were 0.3649 (*p* = 0.0019) and 0.3660 (*p* = 0.0018) (Fig. 3g-h), respectively.

### Difference of Hypometabolism Assessed by AI (AI_1_, AI_2_) in Pre-follow-up and Post-follow-up

**Fig. 4:**
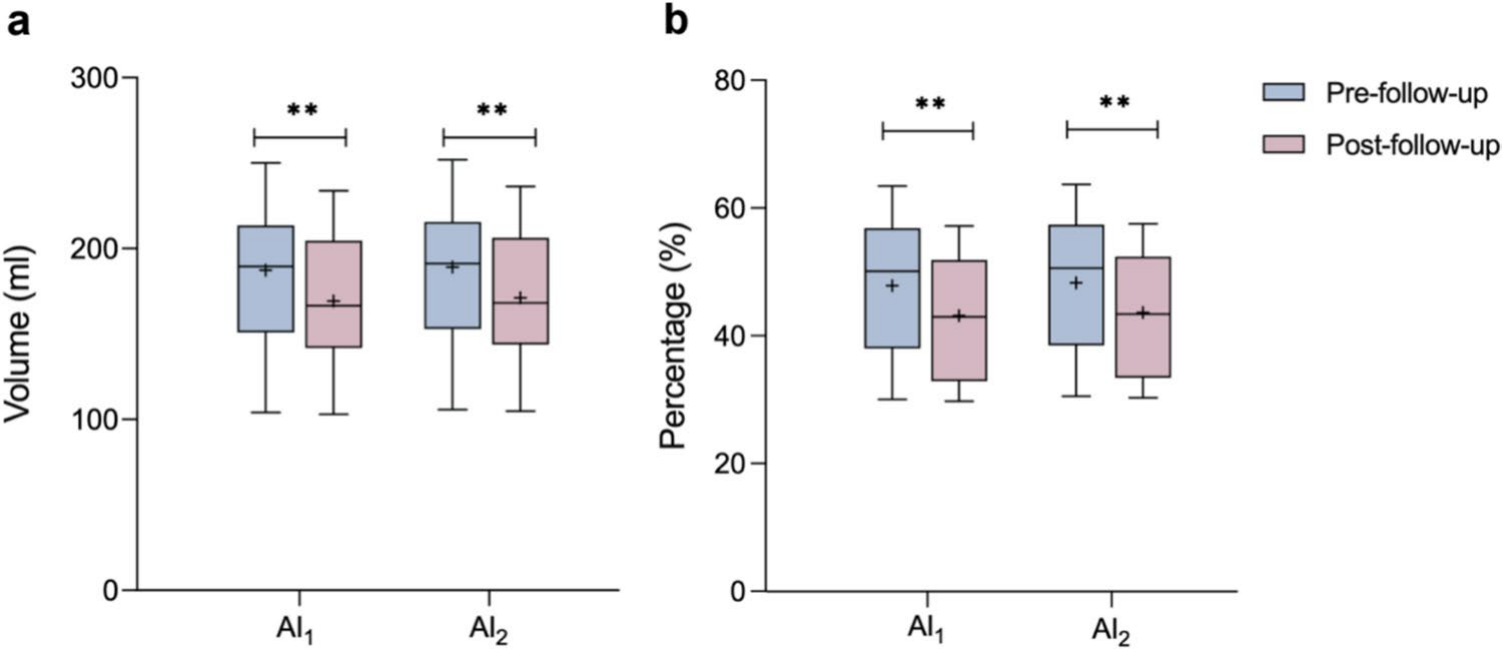
Comparison of the hypometabolic volume **(a)** and percentage **(b)** change of pre-follow-up and post-follow-up patients in AI_1_ and AI_2_ (N = 14). Percentage was presented as the ratio of the hypometabolic volume in affected hemisphere. **p < 0.01

In the follow-up group of fourteen patients, the decreased volume of hypometabolism (without infarction area) as assessed by AI_1_ and AI_2_ methods were 18.07 ± 12.53 and 17.98 ± 12.46, respectively (both *p* < 0.0001; Fig. 4a). And the decreased percentage of hypometabolism as evaluated by AI_1_ and AI_2_ methods were 4.67 ± 3.25 and 4.64 ± 3.23, respectively (both *p* < 0.0001; Fig. 4b). Thus, the reduction of hypometabolic volume and percentage on affected side from AI_1_ method was both greater than that from AI_2_ (both *p* < 0.0001). Table 3 provide a detailed comparison of before and after follow-up evaluations conducted by using AI_1_ and AI_2_ methods. There were significant differences between pre- and post-follow-up in terms of NIHSS (*p* < 0.0047) and mRS (*p* < 0.0001) scores (show in Table 4). The hypometabolism observed in the remaining five patients in follow-up group exhibited an increase, accompanied by a notable improvement in their NIHSS/mRS scores.

**Table 3:** The hypometabolic volume and percentage in the affected hemisphere of the pre- and post-follow-up (N = 14)

	AI_1_					AI_2_			
	Pre-follow-up	Post-follow-up	Change	*p* value		Pre-follow-up	Post-follow-up	Change	*p* value
Volume (ml)	187.35 ± 41.76	169.28 ± 39.64	18.07 ± 12.53	0.0001		189.17 ± 41.63	171.18 ± 39.59	17.98 ± 12.46	0.0001
Percentage (%)	47.84 ± 10.77	43.17 ± 9.84	4.67 ± 3.25	0.0001		48.30 ± 10.71	43.66 ± 9.80	4.64 ± 3.23	0.0001

Mean ± standard deviation was shown for the hypometabolic volume and percentage in the affected hemisphere evaluated by the AI (AI_1_, AI_2_). The p value was shown for the statistically significant difference between the results of pre-follow-up and post-follow-up patients

**Table 4:** The NIHSS and mRS scores of pre-follow-up and post-follow-up patients (N = 14)

Clinical score	Pre-follow-up	Post-follow-up	Change	*p* value
NIHSS	4.5 (1, 9)	1.5 (0, 4)	2 (0, 7)	0.0047
mRS	4 (1, 4)	2 (0, 3)	2 (0, 3)	< 0.0001

The NIHSS and mRS scores were shown as median value (range). The p value was shown for the statistically significant difference between the clinical scores (NIHSS/mRS) from pre-follow-up and post-follow-up patients respectively. NIHSS, National Institutes of Health Stroke Scale; mRS, Modified Rankin Scale

## Discussion

This study aims to compare two AI methods in assessing decreased [^18^F]FDG metabolism for reflecting clinical neurological function in patients with ICVD, and to explore its value in research and clinical settings. The results demonstrated that the volume and percentage of decreased metabolism in the lobes on affected side, excluding the infarct area, attained through AI_2_ calculation consistently greater than those achieved through AI_1_. The correlation between the volume of decreased metabolism in the affected cerebral hemisphere, as calculated by AI_1_ and AI_2_, and the clinical score can be observed. The improved follow-up patients showed more pronounced metabolic improvement as assessed by AI_1_.

### Comparison of Hypometabolism Assessed from AI_1_ and AI_2_ Method

AI_1_ and AI_2_ methods are widely used in studies of ICVD, neurodegenerative diseases and epileptic disorders, mainly for the assessment of structural features within the brain, cerebral metabolism and asymmetry analysis of cerebral blood flow [22–26]. In this study of patients with ICVD, we found that the AI_2_ method consistently yielded larger estimates for the extent of hypometabolism on the affected side, in comparison to the AI_1_ method. It possible to interpret this in terms of the meaning expressed by the structure of the two formulas. Assuming that there is metabolic decrease around the infarction on the affected side, while the metabolism of the corresponding area on the unaffected side is normal, which indicates the absolute difference of metabolism between the two cerebral hemispheres. However, the denominator of the formula of the AI_2_ method is smaller because it is the average of the two sides, which leads to the amplification of the relative difference percentage, so that more voxels satisfy the condition of greater than 10% value. Therefore, the AI_2_ method may exhibit greater sensitivity to hypometabolism. Moreover, in regions with pronounced metabolic reduction on the affected side, such as the temporal and parietal lobes, the smaller denominator (mean value) in the AI_2_ formula will further magnify the relative difference in bilateral metabolism. Consequently, we hypothesize that the exaggerated effect of AI_2_ may be more pronounced in areas with more severe metabolic impairment.

### Comparison of AI_1_ and AI_2_ Methods in Correlating with Clinical Scores

Prior studies have established correlations between AI-assessed cerebral hypometabolism and clinical outcomes in ICVD. [27]. Cui et al. linked preoperative NIHSS scores to AI_1_ values using [^18^F]FDG PET [21], while Yu et al. demonstrated AI_2_'s utility in tracking metabolic changes post-bypass surgery [28]. Sobesky et al. further associated the hypoperfusion volumes assessed by AI with NIHSS and mRS scores [29]. Our findings align with these observations, showing significant correlations between AI1/AI2-quantified hypometabolism and NIHSS/mRS scores, underscoring their clinical monitoring potential.

Notably, AI_1_ exhibited stronger correlations with neurological deficits in temporal/parietal regions compared to AI_2_. According to the cerebral arterial territories research, this distribution characteristic may be related to our subject selection, with cerebral ischemic attributed to the unilateral steno-occlusion attributed to the internal carotid artery or middle cerebral artery [30, 31]. The AI_1_ method employs the unaffected side as a benchmark, thereby quantifying the absolute metabolic loss on the affected side. This metric is directly correlated with clinical scores, including motor and language function. In contrast, AI_2_ may incorporate mild hypometabolic voxels into its calculations due to its smaller denominator. However, these voxels contribute mildly to clinical symptoms, leading to a diminished correlation. These suggest that the AI_1_ method may be more suitable for evaluating the metabolic changes around the infarct area and the clinical status of the unilateral internal carotid artery/middle cerebral artery steno-occlusive disease patients.

While focused on metabolism, our previous work validated AI-based asymmetry analysis for cerebral blood flow using PET/MRI [32], suggesting multimodal applications. Future studies should explore AI-based integration of hemodynamic and metabolic indices to enhance clinical stratification.

### Comparison of Hypometabolism Before and After Follow-up

Sebök et al. [33] reported elevated AI value in ICVD with higher NIHSS/mRS scores, while Zhang H et al. [5] demonstrated that both the significant improvements in neurological function and improvements in brain metabolism shown by [^18^F]FDG PET imaging could be found after treatment. Similarly, our study found improved cerebral metabolism (assessed by AIs) and reduced NIHSS/mRS scores in fourteen follow-up patients, with AI1 showing greater sensitivity to metabolic changes than AI2. However, in five patients with improved NIHSS/mRS scores, hypometabolic areas did not consistently shrink, suggesting a need for closer monitoring and potential treatment adjustments, as neuronal activity correlates with glucose utilization [34, 35].

The quantification of the extent of decreased metabolism around infarct foci due to unilateral internal carotid artery or middle cerebral artery stenosis-occlusion based on AI the intuitive and objective assessment of the decreased metabolic state of the patient's brain tissue by neuroimagers and clinicians alike. For the most common type of ICVD, often caused by unilateral anterior circulation large vessel steno-occlusion, emphasizing metabolic reduction in affected brain tissue is crucial. On the one hand, given that hypometabolism assessed by the AI_1_ method correlates more strongly with clinical scores than the AI_2_ method in this study, we recommend using the AI_1_ method for evaluating cerebral hypometabolism in these patients in clinical practice. In addition, our findings demonstrate that the AI_1_ method can more accurately reflect metabolic changes before and after follow-up. The future research will focus on determining specific cutoff values based on the AI_1_ method to reflect improvements or deteriorations in neurological function, thereby enabling stratified management of ICVD patients in clinical applications. Specifically, This approach to imaging assessment can help guide timely clinical adjustments to further treatment regimens for those assessed to have a poor prognosis, guide close monitoring of the condition, and guide collaborative multidisciplinary clinical management to slow their disease progression, and improve functional outcomes. On another hand, we found that the AI_2_ method appears to be more frequently utilized in the analysis of diseases characterized by bilateral brain tissue lesions, such as ICVD with bilateral multiple cerebral artery steno-occlusion, Alzheimer’s disease and neuropsychiatric disorders through a comprehensive review of existing literature [24–26]. In accordance with varying research objectives and benchmark criteria, we recommend selecting a more suitable research methodology. In the present study, we found that cerebral metabolic evaluation based on AI_2_ approach can lead to an overestimation of the extent of hypometabolism in ICVD patients characterized by unilateral lesions. This overestimation may prompt clinicians to adopt aggressive therapeutic regimens, such as the implementation of cerebral revascularization surgery, frequent or high-intensity neurorehabilitation and prolonged pharmacological interventions. For patients, the unreasonable allocation of medical resources caused by it is likely to increase the psychological burden of patients and cause unnecessary anxiety. In addition, this extra demand for treatment may lead to an increase in the patient's medical costs.

Our study has a few limitations. First, the limited sample size and uniform participant population (unilateral steno-occlusive disease) may restrict broader applicability. Second, the single-center design, while ensuring consistency in imaging protocols and clinical assessments, may introduce selection bias due to regional healthcare variations. In addition, the retrospective nature of the study makes it susceptible to recall bias and information bias. And the rate of loss to follow-up may have impacted the completeness of our data. Future multicenter prospective studies are needed to validate these findings. Moreover, since the follow-up duration in this study was relatively short, the future study will improve the follow-up time (both number and duration) to capture a more comprehensive picture of disease progression and recovery. Additionally, the short follow-up duration should be extended in future studies for a more comprehensive view of disease progression. Lastly, this study employed a semi-quantitative approach. The future research might explore absolute quantitative aspects.

## Conclusions

In summary, the correlation between the clinical scores and the decreased [^18^F]FDG metabolism assessed by AI_1_ method appears more significantly reflecting the improvement of these patient’s clinical condition before and after follow-up. The assessment of cerebral [^18^F]FDG metabolism in patients with unilateral internal carotid/middle cerebral artery steno-occlusion to reflect clinical neurological function using the AI_1_ demonstrated superior performance in comparison to the AI_2_.

## Supplementary Information

Below is the link to the electronic supplementary material.

Supplementary file1 (DOCX 32610 KB)

## Data Availability

All research data and computer codes are available from the corresponding author upon request.
