# Supplementary material for: Comparison of the Correlation Between Cerebral [18F]FDG Metabolism as Assessed by Two Asymmetry Indices and Clinical Neurological Score in Patients with Ischemic Cerebrovascular Disease
Source: Mol Imaging Biol. 2025 Apr 15;27(3):454–64. doi: 10.1007/s11307-025-02002-7 (PMC12162696; doi:10.1007/s11307-025-02002-7)
Supplement: Supplementary file 1 — Supplementary file1 (DOCX 32610 KB) [file 11307_2025_2002_MOESM1_ESM.docx]

**Electronic Supplementary Material**

**Comparison of the correlation between cerebral [^18^F]FDG metabolism as assessed by two asymmetry indices and clinical neurological score in patients with ischemic cerebrovascular disease**

**Journal: Molecular Imaging and Biology**

Yuxin Liang^1,2^, Bixiao Cui^1,2^, Linlin Ye^3^, Bin Yang^4^, Yi Shan^1,2^, Hongwei Yang^1,2^, Lei Ma^1,2^, Miao Zhang^1,2*^, Jie Lu^1,2*^

1.Department of Radiology and Nuclear Medicine, Xuanwu Hospital Capital Medical University, Beijing 100053, China

1. Key Laboratory of Magnetic Resonance Imaging and Brain Informatics, Beijing 100053, China
2. Department of Rehabilitation, Xuanwu Hospital, Capital Medical University, Beijing 100053, China
3. Department of Neurosurgery, Xuanwu Hospital Capital Medical University, Beijing 100053, China

Yuxin Liang and Bixiao Cui contributed equally to this work as co-first authors.

**Corresponding authors:**

Miao Zhang

Changchun Steel 45 #, Xicheng District

Beijing, China, 100037

Tel: 86-13651168079

Email: [zhangmiao_smile@163.com](mailto:zhangmiao_smile@163.com)

Jie Lu

Changchun Steel 45 #, Xicheng District

Beijing, China, 100037

Tel: 86-18911507070

Email: imaginglu@hotmail.com

**Materials and Methods**

*Subjects*

Seventy patients with subacute and chronic ischemic stroke in Xuanwu Hospital, Capital Medical University from March 2018 to August 2023 were retrospectively screened and included according to the following criteria: (1) a confirmed diagnosis of ICVD due to ICA or MCA steno-occlusive; (2) a history of a clinically confirmed stroke of the relevant ICA or MCA territory; and (3) consecutive PET/MR, DWI, computed tomography angiography and magnetic resonance angiography scans. The exclusion criteria were: (1) presence of multiple infarcts on both of the cerebral hemisphere; (2) other neurological disorders that can cause abnormal brain metabolism; and (3) the contraindication for MRI or artefacts on MRI. Following a year of rehabilitation training (non-operative treatment), nineteen patients underwent a repeat follow-up [^18^F]FDG PET/MR scan. The infarcts size and the stroke severities were not used as the criteria for screening patients in this study. The neurological function was assessed using the National Institutes of Health Stroke Scale (NIHSS) and the Modiﬁed Rankin Scale (mRS) scores on admission to hospital. This study was approved by the ethical approval institutional review board of Xuanwu Hospital, Capital Medical University and conducted in accordance with the Declaration of Helsinki. The written informed consents were obtained from all participating patients. Fig. 1 shown the flowchart of the study design.

*PET/MR Image Acquisition*

All scans were collected on a hybrid time of flight (TOF) PET/MR system (Signa, GE Healthcare). Before examination, all subjects fasted for minimum of 6 hours and blood glucose levels were checked to ensure a glycemic level below 8 mmol/L. [^18^F]FDG (3.7 MBq/kg) were injected manually in the median cubital vein, the PET/MR scan started at 50min post-injection. A 19-channel head and neck union coil was used to achieve a high signal-to-noise ratio for PET/MR imaging. Subjects were placed in a supine position to ensure being in the center of the field in view and were instructed to remain calm with their eyes closed.

The [^18^F]FDG PET images were obtained over a period of 10 minutes. The PET data were corrected for attenuation, scatter, random, decay, and dead time. The default attenuation correction sequences (Dixon MR sequences) and MR scans were simultaneously obtained. The Dixon MR sequences was automatically prescribed and acquired as follows: LAVA-Flex (GE Healthcare) axial acquisition, repetition time (TR) = 4 ms, echo time (TE) = 1.7 ms, slice thickness = 5.2 mm, 120 slices, pixel size = 1.95 × 2.93 mm^2^, and acquisition time = 18 s. The corrected PET data were reconstructed using a time-of-flight, point spread function, ordered subset expectation maximization (time of flight - point spread function - office of systems engineering and management, TOF-PSF-OSEM) algorithm with 8 iterations and 32 subsets, and a 3 mm cut-off filter. The resulting pixel size was 1.82 × 1.82 × 2.78 mm^3^.

PET and MR imaging data were simultaneously acquired. The main MRI sequences included the T2 ﬂuid-attenuated inversion recovery (T2-FLAIR) sequence (voxel size = 0.94 × 0.94 × 4.00 mm^3^, TR = 11000 ms, TE = 141 ms, and slices = 32), the diffusion-weighted image (DWI) (b = 0/1000) sequence (voxel size = 1.88 × 1.88 × 4.00 mm^3^, TR = 6189 ms, TE = 74.7 ms, and slices = 32).

*PET/MR Image Preprocessing*

The PET/MR image preprocessing using 3D Slicer Tool (version 5.5.0, <https://www.slicer.org>). Firstly, the skull-stripping was performed on all MRI sequences (T2-FLAIR, DWI, ADC) as well as [^18^F]FDG PET by using HD-BET brain extraction toolkit algorithm[16]. Subsequently, each subject’s [^18^F]FDG PET image was performed partial volume correction (PVC) by using the Van-Cittert algorithm, and the standardized uptake value ratio (SUVR) was calculated using the pons as the reference region[17,18]. Then, the DWI (b=1000), ADC, and PET images were all co-registered to the individual T2-FLAIR image using the General Registration BRAINS algorithm.

*Segmentation Process*

Two experienced neuroradiologists independently and manually delineated the cerebral infarction regions based on the T2-FLAIR sequence. For cases of disagreement, a consensus was reached in a separate session. The individual brain PET image was subdivided into 83 regions by using an automated labeling system, neuroparc (https://github.com/neurodata/neuroparc)[19]. And the bilateral frontal, temporal, parietal, and occipital regions were left behind in preparation for the next step of analysis.

*Asymmetry Index (AI) Measurements*

AI is the method used to measure the asymmetry of a distribution. The [^18^F]FDG PET images were converted into maps that represented the standardized uptake value ratio (SUVR) for each voxel. The SUVR was calculated by dividing the tissue concentration of radioactivity (kBq/mL) in the region of interest by the mean activity concentration in a reference region. The AI was calculated in the individual brain map after removing all cerebral infarctions on both ipsilateral and contralateral sides to assess the left-right asymmetry on SUVR value. Two formulas for evaluating asymmetry, named AI_1_ and AI_2_, were computed based on the following two equations (1) (2) respectively[20]:

$$AI_{1}=\frac{SUVR_{contralateral}-SUVR_{ipsilateral}}{SUVR_{contralateral}}\text{×}100\%$$

$$AI_{2}=\frac{2\times(SUVR_{contralateral}-SUVR_{ipsilateral})}{SUVR_{contralateral}+SUVR_{ipsilateral}}\text{×}100\%$$

where contralateral (ipsilateral) represents the affected side (unaffected side). Subsequently, the volume of decreased metabolism on affected side was defined as those voxel-wise AI value higher than 10%[21]. And we calculated the percentage change in frontal, temporal, parietal, occipital regions and cerebral hemisphere on affected side compared to the unaffected side.

*Statistical Analysis*

All statistical analyses were conducted using IBM SPSS Statistics for Windows, version 27.0 (IBM). Categorical variables were expressed as percentages. Normally distributed metric variables were expressed as mean ± standard deviation (SD). Non-normally distributed variables were expressed as median (range). Bland-Altman analysis was used for analyzing the quantitative agreement between AI_1_ and AI_2_ methods. The mean of the absolute values of the differences and the limits of agreement were calculated for each patient. Metabolic differences across the AI calculation methods were assessed using Paired T-tests. The statistical significance was determined at *p* value<0.05. The correlation between the AI assessments and NIHSS/mRS score were analyzed using Spearman’s rank correlation. The data before and after the follow-up were all analyzed.

**Results**

*Patient characteristic*

A total of 70 patients, comprising 51 males (72.86%) with a mean age of 52 ± 11 years, presenting with unilateral internal carotid artery and middle cerebral artery steno-occlusive disease, underwent a [^18^F]FDG PET/MR scan. The NIHSS and mRS scores were recorded for each patient. Additionally, nineteen of these patients (including 10 males (52.63%) with a mean age of 51 ± 14 years at pre-follow-up and 53 ± 13 years at post-follow-up) underwent a repeated follow-up [^18^F]FDG PET/MR scan and were reassessed using the NIHSS and mRS after a year of rehabilitation training. Fig. 2 illustrates the exemplary imaging of one participant both before and after the follow-up period. Table 1 showed the detailed demographic characteristics.

*The hypometabolic state on affected side as assessed by AI_1_ and AI_2_*

We found the hypometabolic volume and percentage in individual lobes and entire hemisphere on affected side (without infarction area) obtained from AI_2_ method were all greater than that of AI_1_. The volume of hypometabolism was calculated as 176.66 ± 36.09 vs. 178.68 ± 36.11 (*p* < 0.0001) for AI_1_ and AI_2_ on the affected cerebral hemisphere, resulting in the percentage of hypometabolism of 43.52 ± 8.72 vs. 44.02 ± 8.69 (*p* < 0.0001). The volume and percentage of hypometabolism observed in frontal, temporal, parietal, and occipital lobes yielded comparable outcomes (all *p* < 0.0001) (shown in Table 2). Bland-Altman analysis showed a close degree of agreement between measurements of metabolic reduced volume and percentage between AI_1_ and AI_2_ methods (Suppl. Fig.1).

*Correlation between the hypometabolic volume as evaluated by AI (AI_1_, AI_2_) and the NIHSS/mRS score*

A significant correlation between the hypometabolic volume (without infarction area) and the NIHSS score can be observed while the correlation coefficients obtained from the AI_1_ method were all higher than those from the AI_2_ (Fig.3 and Suppl. Fig.2). The correlation coefficients in temporal lobe obtained by the AI_1_ and AI_2_ methods were 0.3403 (*p* = 0.0039) and 0.3393 (*p* = 0.0041) (Suppl. Fig.2a-b) while in parietal lobe were 0.3076 (*p* = 0.0096) and 0.3052 (*p* = 0.0102) (Suppl. Fig.2e-f), respectively. And the correlation coefficients in whole affected hemisphere obtained by the AI_1_ and AI_2_ methods were 0.3010 (*p* = 0.0113) and 0.2942 (*p* = 0.0134) (Fig.3a-b), respectively.

Similar results demonstrated that the hypometabolic volume (without infarction area) obtained by the AI_1_ exhibited a stronger correlation with mRS than that obtained from AI_2_ (Fig.3 and Suppl. Fig.2). The correlation coefficients in temporal lobe obtained by the AI_1_ and AI_2_ methods were 0.2751 (*p* = 0.0212) and 0.2745 (*p* = 0.0215) (Suppl. Fig.2c-d) while in parietal lobe were 0.2885 (*p* = 0.0154) and 0.2825 (*p* = 0.0178) (Suppl. Fig.2g-h), respectively. And the correlation coefficients in whole affected hemisphere obtained by the AI_1_ and AI_2_ methods were 0.3010 (*p* = 0.0113) and 0.2942 (*p* = 0.0134) (Fig.3c-d), respectively.

*Correlation between the hypometabolic percentage as evaluated by AI (AI_1_, AI_2_) and the NIHSS/mRS score*

The significant correlation between the hypometabolic percentage (without infarction area) and the NIHSS score can be observed while the correlation coefficients obtained from the AI_1_ method were all higher than those from the AI_2_ (Suppl. Fig.3 and Suppl. Fig.4). The correlation coefficients in temporal lobe were 0.3550 (*p* = 0.0026) and 0.3516 (*p* = 0.0028) (Suppl. Fig.3e-f) while in parietal lobe were 0.4887 (*p* < 0.0001) and 0.4866 (*p* < 0.0001) (Suppl. Fig.4a-b), respectively.

The higher correlation in AI_1_ could also be found between the hypometabolic percentage and the mRS score (Suppl. Fig.3 and Suppl. Fig.4). The correlation coefficients in temporal lobe obtained by the AI_1_ and AI_2_ methods were 0.2917 (*p* = 0.0143) and 0.2874 (*p* = 0.0159) (Suppl. Fig.3g-h) while in parietal lobe were 0.4504 (*p* < 0.0001) and 0.4485 (*p* < 0.0001) (Suppl. Fig.4c-d), respectively.

The hypometabolic percentage in whole affected hemisphere as assessed by AI_2_ method demonstrated a slightly stronger correlation with NIHSS/mRS scores than that obtained from AI_1_ (Fig.3). Specifically, the correlation coefficients with NIHSS score obtained by the AI_1_ and AI_2_ methods were 0.4076 (p = 0.0005) and 0.4094 (p = 0.0004) (Fig. 3e-f) while with mRS score were 0.3649 (*p* = 0.0019) and 0.3660 (*p* = 0.0018) (Fig. 3g-h), respectively.

*Difference of hypometabolic state assessed by AI_1_ and AI_2_ in pre-follow-up and post-follow-up*

In the follow-up group of fourteen patients, the decreased volume of hypometabolism (without infarction area) as assessed by AI_1_ and AI_2_ methods were 18.07 ± 12.53 and 17.98 ± 12.46, respectively (both *p* < 0.0001; Fig. 4a). And the decreased percentage of hypometabolism as evaluated by AI_1_ and AI_2_ methods were 4.67 ± 3.25 and 4.64 ± 3.23, respectively (both *p* < 0.0001; Fig. 4b). Thus, the reduction of hypometabolic volume and percentage on affected side from AI_1_ method was both greater than that from AI_2_ (both *p* < 0.0001). Table 3 provide a detailed comparison of before and after follow-up evaluations conducted by using AI_1_ and AI_2_ methods. There were significant differences between pre- and post-follow-up in terms of NIHSS (*p* < 0.0047) and mRS (*p* < 0.0001) scores (show in Table 4). The hypometabolism observed in the remaining five patients in follow-up group exhibited an increase, accompanied by a notable improvement in their NIHSS/mRS scores.


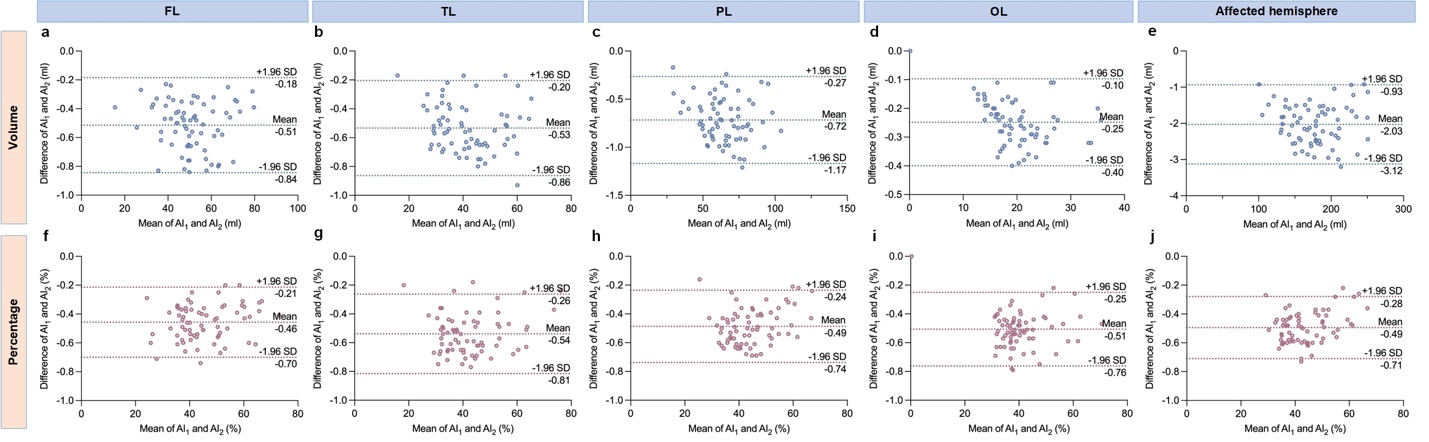


**Supplementary Figure 1.** The Bland-Altman plot of the hypometabolism assessed by AI_1_ and AI_2_ methods. Mean absolute differences and limits of agreement (±1.96SD) are shown for each lobe and hemisphere on affected side. AI, asymmetry index. FL, Frontal lobe; TL, Temporal lobe; PL, Parietal lobe; OL, Occipital lobe. Bland-Altman plot of hypometabolic volume in FL (−0.51 mL (±0.33 mL); Figure a), TL (−0.53 mL (±0.33 mL); Figure b), PL (−0.72 mL (±0.45 mL); Figure c), OL (−0.25 mL (±0.15 mL); Figure d) and affected hemisphere (−2.03 mL (±0.09 mL); Figure e) demonstrate the close agreement for AI_1_ and AI_2_ methods. Bland-Altman plot of hypometabolic percentage in FL (−0.46 % (±0.24 %); Figure f), TL (−0.54 % (±0.28 %); Figure g), PL (−0.49 % (±0.25 %); Figure h), OL (−0.51 % (±0.26 %); Figure i) and affected hemisphere (−0.49 % (±0.22 %); Figure j) also demonstrate the close agreement for AI_1_ and AI_2_ methods.

**
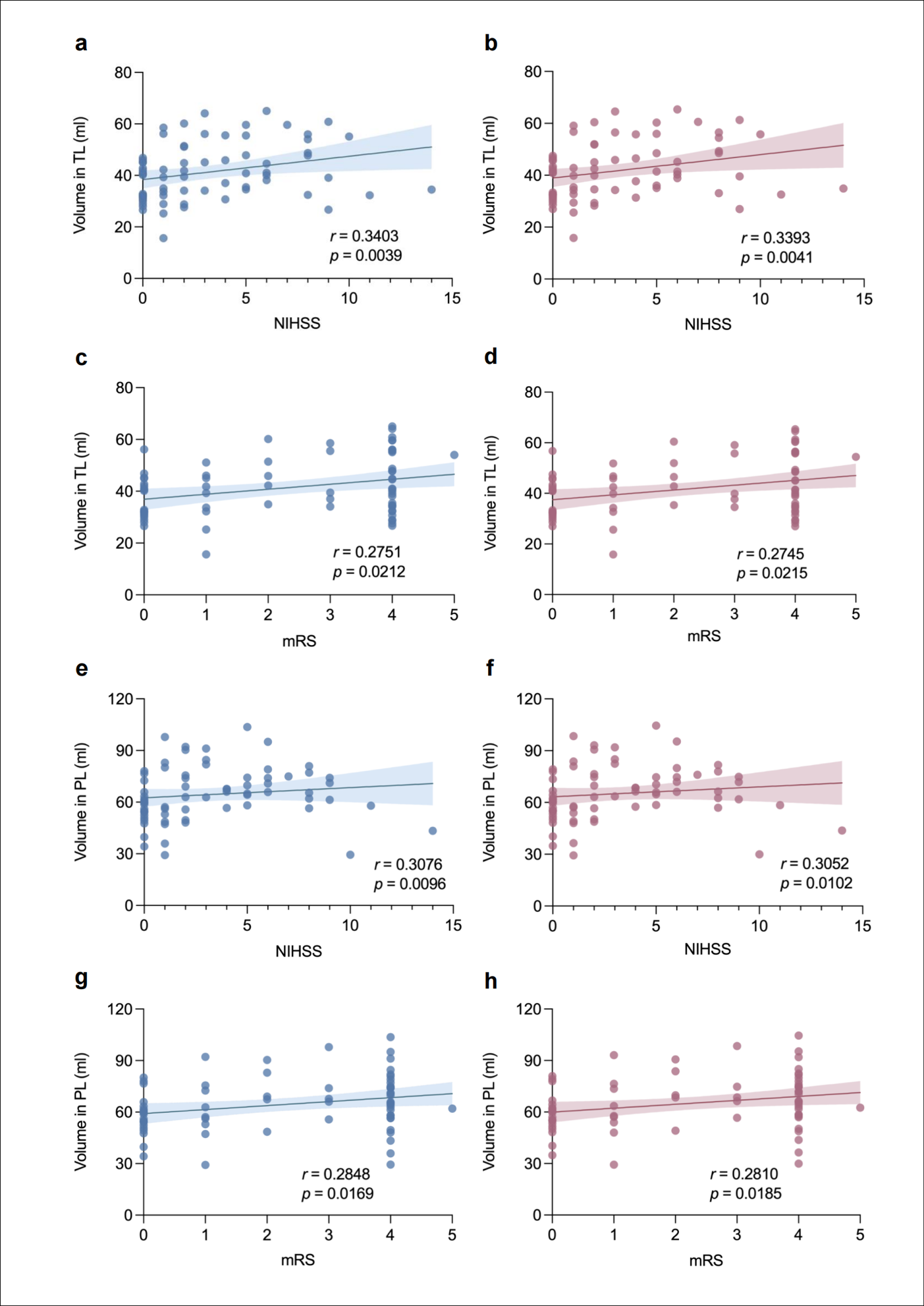
**

**Supplementary Figure 2.** The correlation between the hypometabolic volume in different regions in AI_1_ and AI_2_ with the clinical scores(NIHSS/mRS) (N=70). NIHSS, National Institutes of Health Stroke Scale; mRS, Modified Rankin Scale. TL, Temporal lobe; PL, Parietal lobe. **(a)** Correlation between the volume in temporal lobe in AI_1_ and NIHSS score. **(b)** Correlation between the volume in temporal lobe in AI_2_ and NIHSS score. **(c)** Correlation between the volume in temporal lobe in AI_1_ and mRS score. **(d)** Correlation between the volume in temporal lobe in AI_2_ and mRS score. **(e)** Correlation between the volume in parietal lobe in AI_1_ and NIHSS score. **(f)** Correlation between the volume in parietal lobe in AI_2_ and NIHSS score. **(g)** Correlation between the volume in parietal lobe in AI_1_ and mRS score. **(h)** Correlation between the volume in parietal lobe in AI_2_ and mRS score.


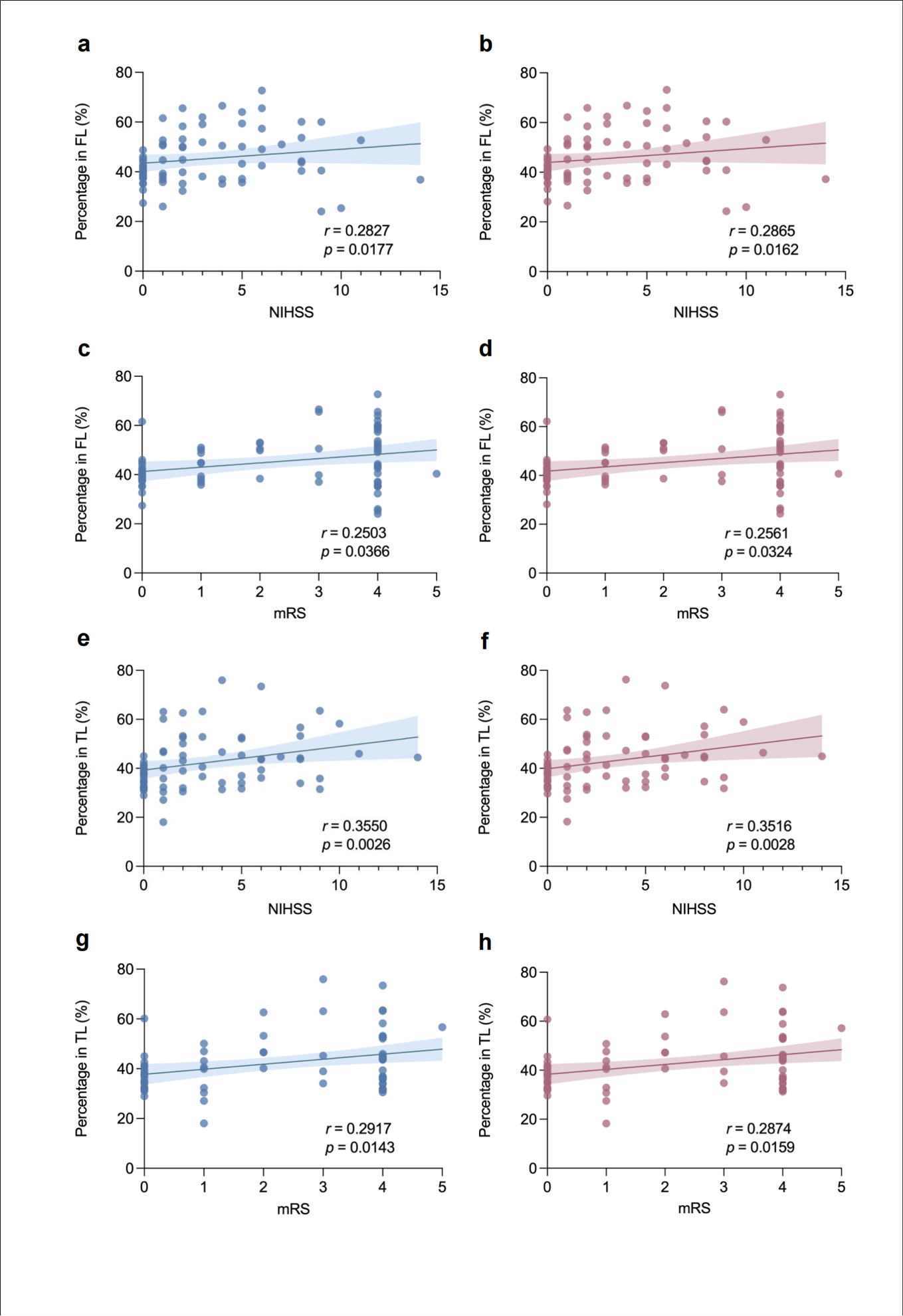


**Supplementary Figure 3.** The correlation between the hypometabolic percentage in different regions in AI_1_ and AI_2_ with the clinical scores(NIHSS/mRS) (N=70). NIHSS, National Institutes of Health Stroke Scale; mRS, Modified Rankin Scale. FL, Frontal lobe; TL, Temporal lobe. **(a)** Correlation between the percentage in frontal lobe in AI_1_ and NIHSS score. **(b)** Correlation between the percentage in frontal lobe in AI_2_ and NIHSS score. **(c)** Correlation between the percentage in frontal lobe in AI_1_ and mRS score. **(d)** Correlation between the percentage in frontal lobe in AI_2_ and mRS score. **(e)** Correlation between the percentage in temporal lobe in AI_1_ and NIHSS score. **(f)** Correlation between the percentage in temporal lobe in AI_2_ and NIHSS score. **(g)** Correlation between the percentage in temporal lobe in AI_1_ and mRS score. **(h)** Correlation between the percentage in temporal lobe in AI_2_ and mRS score.


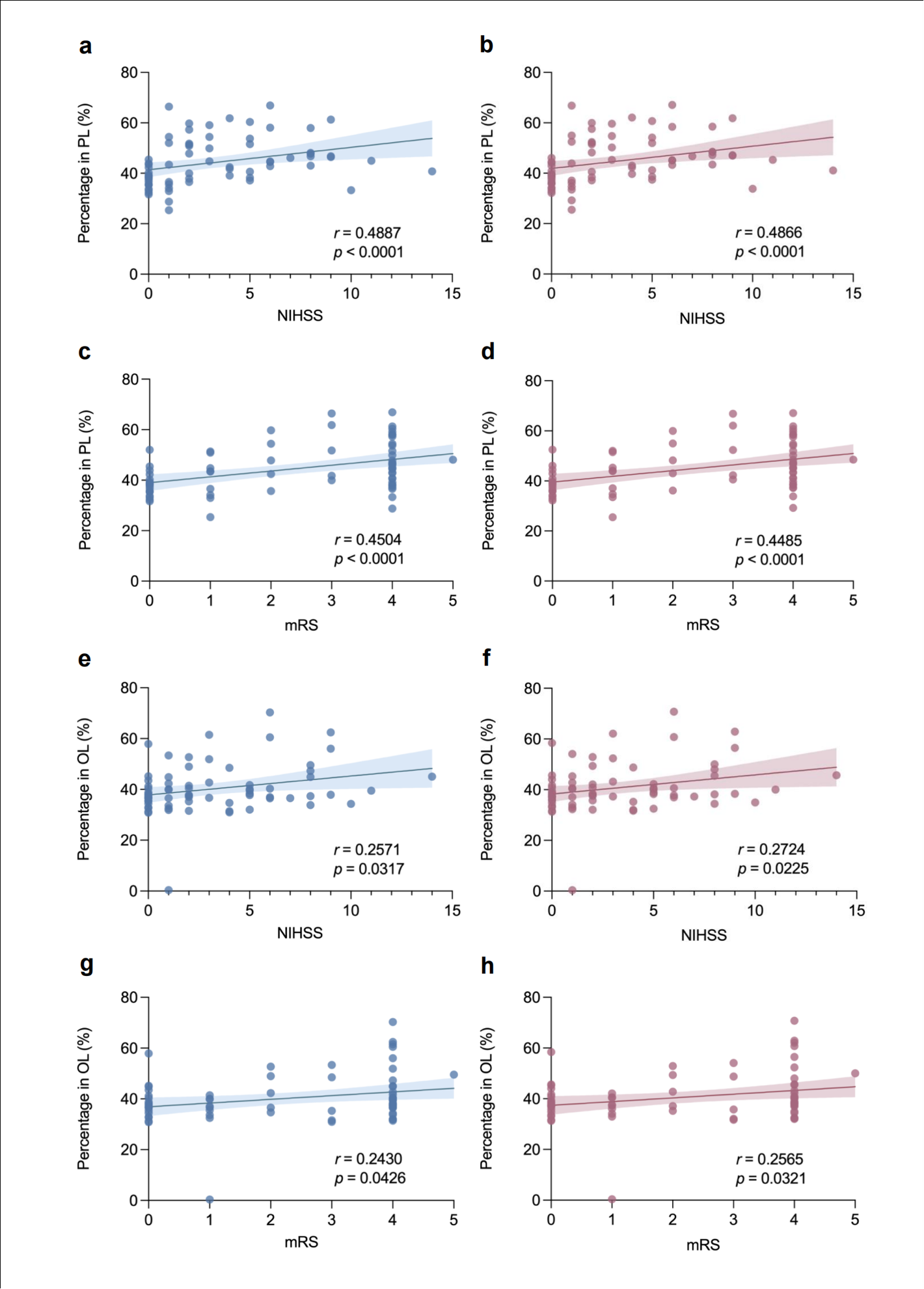


**Supplementary Figure 4.** The correlation between the hypometabolic percentage in different regions in AI_1_ and AI_2_ with the clinical scores(NIHSS/mRS) (N=70). NIHSS, National Institutes of Health Stroke Scale; mRS, Modified Rankin Scale. PL, Parietal lobe; OL, Occipital lobe. **(a)** Correlation between the percentage in parietal lobe in AI_1_ and NIHSS score. **(b)** Correlation between the percentage in parietal lobe in AI_2_ and NIHSS score. **(c)** Correlation between the percentage in parietal lobe in AI_1_ and mRS score. **(d)** Correlation between the percentage in parietal lobe in AI_2_ and mRS score. **(e)** Correlation between the percentage in occipital lobe in AI_1_ and NIHSS score. **(f)** Correlation between the percentage in occipital lobe in AI_2_ and NIHSS score. **(g)** Correlation between the percentage in occipital lobe in AI_1_ and mRS score. **(h)** Correlation between the percentage in occipital lobe in AI_2_ and mRS score.
